# Family resources study: part 1: family resources, family function and caregiver strain in childhood cancer

**DOI:** 10.1186/1447-056X-10-14

**Published:** 2011-10-31

**Authors:** Avegeille T Panganiban-Corales, Manuel F Medina

**Affiliations:** 1 Department of Family and Community Medicine University of the Philippines-Philippine General Hospital Taft Avenue, Ermita, Manila Philippines

**Keywords:** family resources, caregiver strain, caregiver stress, caregiver burden, family function, family's coping mechanism, family resources assessment

## Abstract

**Background:**

Severe illness can disrupt family life, cause family dysfunction, strain resources, and cause caregiver burden. The family's ability to cope with crises depends on their resources. This study sought to assess families of children with cancer in terms of family function-dysfunction, family caregiver strain and the adequacy of family resources using a new family resources assessment instrument.

**Methods:**

This is a cross-sectional study involving 90 Filipino family caregivers of children undergoing cancer treatment. This used a self-administered questionnaire composed of a new 12-item family resources questionnaire (SCREEM-RES) based on the SCREEM method of analysis, Family APGAR to assess family function-dysfunction; and Modified Caregiver Strain Index to assess strain in caring for the patient.

**Results:**

More than half of families were either moderately or severely dysfunctional. Close to half of caregivers were either predisposed to strain or experienced severe strain, majority disclosed that their families have inadequate economic resources; many also report inaccessibility to medical help in the community and insufficient educational resources to understand and care for their patients. Resources most often reported as adequate were: family's faith and religion; help from within the family and from health providers. SCREEM-RES showed to be reliable with Cronbach's alpha of 0.80. There is good inter-item correlation between items in each domain: 0.24-0.70. Internal consistency reliability for each domain was also good: 0.40-0.92. Using 2-point scoring system, Cronbach's alpha were slightly lower: full scale (0.70) and for each domain 0.26-.82. Results showed evidence of association between family resources and family function based on the family APGAR but none between family resources and caregiver strain and between family function and caregiver strain.

**Conclusion:**

Many Filipino families of children with cancer have inadequate resources, especially economic; and are moderately or severely dysfunctional. Many caregivers are predisposed to caregiver strain or are already experiencing severe strain. To provide appropriate care for these families, physicians should regularly assess family function, resources and strain experienced by caregivers. The SCREEM-RES questionnaire used in this study is a helpful and reliable instrument to assess adequacy of family resources.

## Background

Approximately 144 per 1 million Filipino children and adolescents are diagnosed with cancer each year in Philippines [1]. The diagnosis of cancer in a child can send a family into crisis. It not only disrupts the child's life but the family's life as well - as family members try to adjust their roles, interactive patterns and relationships inside and outside of the family. The crisis drastically changes 'normal' routines of family interactions as the family attempts to cope. The family's ability to cope with a medical crisis such as cancer in a child depends on their resources. In the Philippines, many families may lack financial and other resources to deal with childhood cancer.

According to Barakat, Marmer and Schwartz [2], family function is central to the quality of life in children while under treatment for cancer; it may promote positive outcomes for them. Moreover; "...families of children and adolescents with cancer rate themselves as less cohesive and more conflicted than do families of healthy children" [3]. Family function measures the extent to which a family works as a unit; it denotes the family's ability to cope and adjust to different situations. Screening for family functioning is important to identify strengths that can serve as buffers in coping with stressors [4]. This can be done with the use of family APGAR originally designed by Smilkstein [5]. A Filipino version of the tool has been used and validated in the Philippines since 1992; its results reflect a family member's perception of and satisfaction with the functional state of his family. It is one of the most common tools used by family physicians for assessing family function in the clinical setting.

Childhood cancer causes severe disruptions in family life and results in many stressors of varying duration, certainty and impact [6-9]. The effects of childhood cancer are often felt by the entire family, especially the family caregiver. Moreover, because children are not legally competent, most decisions about cancer and its management are made by the adult family members and caregivers. Childhood cancer can cause significant psychosocial distress on adult family members and caregivers.

Manne et. al. [10] found that moderate to severe depressive symptoms in parents did not improve 6 months after diagnosis. Sloper et. al. [11] also found that psychological distress in parents remained high at 18 months after diagnosis.

Caregiver strain measures stress or burden due to the physical, personal, emotional and financial stress incurred by a caregiver as a result of, or in relation to, his/her caregiving role/s. In the study done by Beck and Lopes among caregivers of children with cancer in 2007, 78% of the caregivers had "caregiver role strain" and 100% presented risk for "caregiver role strain" [12]. It is therefore important to identify strain among caregivers to prevent adverse psychosocial problems on the family caregiver.

According to Dr. Gabriel Smilkstein [5,13] the family's ability to adapt to or cope with crisis depends on their resources. In the Philippines, a popular method used by family physicians to assess family resources is the SCREEM Method of Analysis which was developed by Smilkstein [5] to determine family's capacity to participate in the provision of health care or to cope in times of crisis. Use of this tool results in a narrative description of the social, cultural, religious, economic, educational and medical resources, for which its acronym stands for, as it relates to possible source of help or hindrance to the provision of care for patients.

At present, there is a need for reliable and valid Filipino instruments to assess the adequacy of family resources. The specific objectives of the study were: 1) to determine family function using the Filipino Family APGAR; 2) to measure the family caregiver strain utilizing the Modified Caregiver Strain Index; 3) to evaluate family resources using a new questionnaire based on the six domains of the SCREEM method of analysis- social, cultural, religious, economic, education and medical; 3) to determine the relationship between family resources, family function and caregiver strain; and 4) to determine the reliability of the new family resources questionnaire in terms of internal consistency among pediatric cancer patients.

## Operational Definitions

• family resources - means that can be used by the family to cope with difficult situations; include the following resources:

social resources - strong social support network which may include spouse, children, parents, siblings, neighbors, co-workers and others

cultural resources - cultural values which can influence an individual or family's ability to care for the sick and cope with stress e.g. optimism, familialism, approach vs avoidance style, etc.

religious resources - spiritual beliefs, practices and support services

economic resources - family's income and savings

educational resources - level of formal education attained by an individual which allows him to understand the patient's condition and give him appropriate care

medical resources - acessibility to medical facilities and adequacy of help from healthcare providers

These resources will be measured by the use of SCREEM-RES, a newly developed family resources questionnaire developed by the staff of the section of Supportive Palliative Hospice Medicine of the University of the Philippines-Philippine General Hospital.

• caregiver strain - physical, personal, emotional and financial stress incurred by a caregiver as a result of, or in relation to, his/her caregiving role/s. This will be measured by the Modified Caregiver strain index

• family function - measures the extent to which a family works as a unit; it denotes the family's ability to cope and adjust to different situations based on 5 components: adaptation, partnership, growth, affection and resolve; the Filipino Family APGAR will be used to measure this

## Methods

### Sample and Data Collection

This is a cross sectional study involving a non-randomized convenience sample of 90 adult family caregivers of children with cancer who were being seen at the pediatric cancer clinic of a tertiary government hospital in the Philippines. The study included family caregivers who were 18 years old and above, and able to understand procedures and follow instructions, read and understand Filipino language. After the consent was obtained, a self-administered questionnaire was given. The socio-demographic profile was also obtained through the questionnaire.

The study used convenience sampling, which is useful for descriptive and correlation studies in relatively new areas of investigation [14]. Several motivations to use this method are the following: when it is difficult to create a list for making a random selection, when the objectives of the study do not require exact results, such as in biopsychosocial surveys; or when the researcher is interested in a population of which only a few cases may be available for study and these then must serve as the sample of the population, such as families of children with cancer.

### Data Collection Tools and Instruments

#### Filipino Family APGAR

Family function measures the extent to which a family works as a unit; it denotes the family's ability to cope and adjust to different situations based on 5 components: adaptation, partnership, growth, affection and resolve. The Filipino family APGAR is a translated Filipino version of Smilkstein's family APGAR. Its use has been analyzed in the Philippines by "Drs. L Cabahug and A. Pineda, Jr in their study "Family APGAR: Its Validation Among Filipino Families Emergency Room, Out Patient Department Sto. Tomas University Hospital, January to April, 1992." This research showed that the translated Filipino APGAR has a good reliability index as its contents approximated the Smilkstein's English version. Like the original family APGAR, the Filipino family APGAR consists of statements on five (5) parameters of family functioning: Adaptability, Partnership, Growth, Affection and Resolve. The family member's response is based on the frequency of feeling satisfied with each of the five parameters using a 3-point scale ranging from 0 (hardly ever) to 2 (almost always). The scale is scored by summing the values for the five items for a total score that can range from 0-10. A score of 0-3 denotes a severely dysfunctional family, 4-7 moderately dysfunctional family and 8-10 highly functional family [5,15].

#### Modified Caregiver Strain Index (MCSI)

Caregiver strain measures stress or burden due to the physical, personal, emotional and financial stress incurred by a caregiver as a result of, or in relation to, his/her caregiving role/s. The Modified Caregiver Strain Index (MCSI) for Filipino caregivers is an 11-item questionnaire that deals with four major areas of burden - physical, personal, emotional and financial. Each item can be answered by a three-point Likert scale. Summing the scores in all of the items will determine if a caregiver has strain. A score of ≤ 23 is normal, 24-28 shows propensity for strain and ≥ 29 signifies severe caregiver strain [16,17].

#### SCREEM Family Resources Survey (SCREEM-RES)

Family resources are the means that can be used by the family to cope with difficult situations; these include social, cultural, religious, economic and medical resources. A new family resources questionnaire developed by Dr. Medina and the Section of Supportive Palliative and Hospice Medicine (SPHM), University of the Philippines - Philippine General Hospital was used to assure homogeneity in the data that will be collected regarding the subject's perception of the adequacy of the family's resources [Medina, M. (2010). SCREEM Based Family Resources Survey. Unpublished Manuscript.]. This tool was formulated by the authors to create a valid and reliable measure of family resources that would be useful in clinical practice and research. It was based on the: 1) SCREEM method of analysis by Smilkstein [5,13] which has been used in the local setting to assess family's capacity to participate in the provision of health care or to cope with crisis, as well as from 2) reviews of published surveys, and also 3) current opinion and experience of specialists of the Section of SPHM in the use of the original SCREEM Method of Analysis. This instrument is a brief 12 item questionnaire which includes all the six original SCREEM domains, and contains 2 items per domain. Family caregivers are asked to choose one of the following responses: strongly disagree ("lubos na hindi sumasang-ayon"), disagree ("hindi sumasang-ayon"), agree ("sumasang-ayon"), and strongly agree ("lubos na sumasang-ayon").

### Data Analysis

Descriptive statistics was done using means for continuous variables; frequencies using categorical variables. For continuous variables, tests of differences between means and independent groups were done to determine difference. Reliability analysis was done by measuring inter-item correlation, item-total correlation, and Cronbach's alpha.

For preliminary test for association, the study used simple dichotomous categories for the SCREEM Questionnaire. Each item was scored on a 0 - 3 basis using the following key: Strongly Disagree = 0, Disagree = 1, Agree = 2, and Strongly Agree = 3. The scores for all of the items, domains and entire SCREEM-RES were summed resulting to scores of 0-3 for each item, 0-6 for each domain and 0-36 for the entire SCREEM-RES. The scores were further grouped to determine inadequacy or adequacy of resources. Inadequate resources for each item have scores of 0 to 1, for each domain 0 to 3 and for the total SCREEM-RES 0-18. On the other hand, adequate resources are signified by the following scores, 2 to 3 for each item, 4 to 6 for each domain and 19 to 36 for the whole SCREEM-RES.

Chi-square test was used to determine association between family resources, family function and caregiver strain. Exact tests were used when indicated, such as when expected frequencies were lower than 5. Cramer's V was used to determine the strength of association; and it increases from 0 to 1 as the strength of association increases.

An exploratory factor analysis was also done to examine the structure of relationships between the items of the family resources questionnaire. All statistical analysis in the study was done using SPSS. For the study, level of significance was set at 0.05.

## Results

### Demographics

A total of 90 Filipino family caregivers of Filipino children with cancer took part in the study. Table 1 shows the family caregivers' demographics. Caregivers' ages ranged from18-65 years old with a mean age of 36 years. Most of them were able to reach high school (62%) and are the patients' mothers (75%).

**Table 1:** Family Caregiver Demographics

	Number of Caregivers	Percent (%)
Age of Family Caregiver (in years)	Mean = 35.67 (SD = 10.65)	Median = 35.00
**Age Group of Caregiver**		
Young adult (18-35)	44	49.4
Middle Adulthood (35-60)	43	48.3
Late adulthood (60)	2	2.2
**Sex**		
Male	15	16.7
Female	75	83.3
**Educational background**		
Elementary	6	6.7
High school	55	61.8
College	27	30.3
**Relationship to Patient**		
Mother	65	74.7
Father	14	16.1
Grandmother	3	3.4
Sibling	3	3.4
Aunt	2	2.3

Table 2 shows the patients' demographics. Mean age was 7-8 years (SD = 5.37). Most of the children have hematologic malignancies and most have been undergoing treatment for 1 year or less.

**Table 2:** Patient Demographics.

	Mean	Median
**Age of Patient (in years)**	7.9 (SD = 5.37)	7.0
**Age of Patient at Diagnosis**	5.6 (SD = 4.93)	4.0
**Duration of Treatment**	1.6 (SD = 2.23)	1.0
**Cancer Type**	**Number of Patients**	**Percent (%)**
**Hematologic**	60	67.0
**Non hematologic**	28	31.0
**missing**	2	0.3

### Family Function and Caregiver Strain

**Figure 1:**
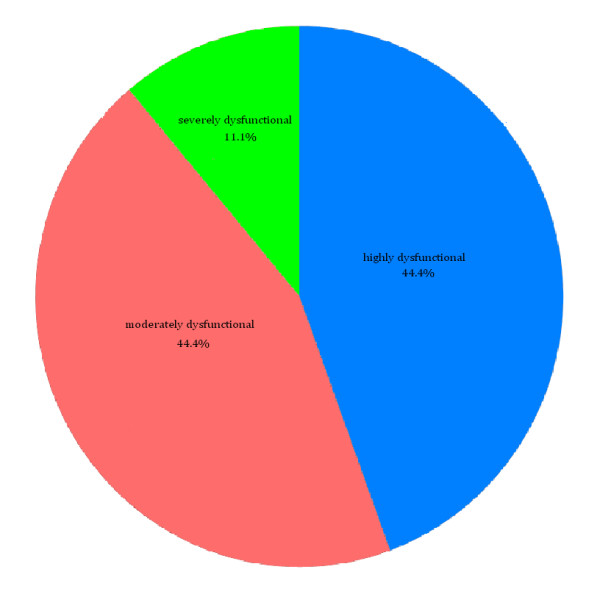
**Caregivers' Family APGAR**.

Based on the results of the Family APGAR, illustrated in Figure 1, 44.4% of family caregivers reported their families as being highly functional, 44.4% moderately dysfunctional and 11.1% are severely dysfunctional.

**Figure 2:**
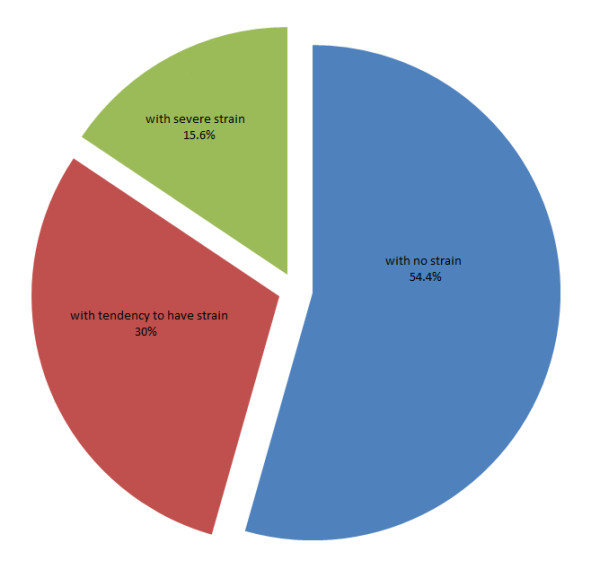
**Caregivers' Strain**.

It is shown in Figure 2 that 54.4% of the caregivers were without caregiver strain, 30.0% were with predisposition to caregiver strain and 15.6% were with severe caregiver strain.

### Family Resources

Table 3 shows the caregivers responses regarding the availability of family resources using the family resources questionnaire. Inadequate family income and family savings were reported by 83.3% and 80.9% of family caregivers respectively. Difficult access to medical help in the community was reported by 38.9%. 23.3% felt that their education was inadequate to understand information about their patients' disease. Adequate faith and religion and adequate help from religious groups were reported by 97.8% and 84.4% of family caregivers respectively. Sufficient help within the family and community were reported by 94.5% and 82.2% of family caregivers in that order. 91.1% and 88.9% reported that their culture strengthens their family and the culture of helping and sharing is adequate in their community correspondingly.

**Table 3:** Percentage of Family Caregivers Reporting on the Adequacy of Family Resources.

		Percentage Response (%)	
Resource Domains	Domain Items	Agree that Resources are Adequate	Disagree that Resources are Adequate
Social	Help within family	94.5	5.5
	Help from community	82.2	17.8
**Cultural**	**Culture strengthens family**	**91.1**	**8.9**
	**Culture of helping and cooperation**	**88.9**	**11.1**
Religious	Faith & Religion helps family	97.8	2.2
	Help from Religious groups	84.4	15.6
**Economic**	**Family Savings**	**19.1**	**80.9**
	**Family Income**	**16.7**	**83.3**
Educational	Adequate to understand illness	76.7	23.3
	Adequate to care for patient	83.3	16.7
**Medical**	**Access to medical care in community**	**61.1**	**38.9**
	**Help from health care providers**	**96.7**	**3.3**

### Reliability Analysis

Cronbach's alpha (α) for the entire scale was 0.80, using the original scores from 0 to 3 for each item, indicating good internal consistency reliability. There is a wide range of values in the inter-item correlation matrix (-0.13 to 0.69) for all the items which is consistent with the highly multidimensional nature of the scale. However, there is good inter-item correlation between items in each domain: social (0.27), cultural (0.51), religious (0.35), economic (0.70), educational (0.67), and medical (0.24). Internal consistency reliability, α, for each domain were: social (0.61), cultural (0.77), religious (0.60), economic (0.92), educational (0.78), and medical (0.40).

In preparation for the planned tests for independence and association, further analysis was done using a simplified dichotomous scoring system. Item scores were grouped into two (0-1 = inadequate, 2 - 3 = adequate). Using this scoring system, item analysis and reliability results were lower compared to analysis using the original (0 to 3) scores. Internal consistency remained good (α = 0.70). Internal consistency reliability, α. for each domain were: social (0.38), cultural (0.67), religious (0.39), economic (0.82), educational (0.80), and medical (0.26). Inter-tem correlations for each domain and for the entire instrument remained unchanged.

### Analysis for Independence and Association

Tests for independence and association were done using dichotomous categories for family resources and standard categories recommended for the Family APGAR (3 categories) and the MCSI (3 categories). Significant associations were found between family function based on the Family APGAR and the adequacy of the following domains of family resources: social (chi square (2) = 18.440, exact p < 0.001; p = 0.001, Fisher's Exact Test, FET), cultural (chi square (2) = 13.127, exact p = 0.002; p = 0.003, FET), and medical (chi square (2) = 8.291, exact p = 0.015; p = 0.017, FET). Computed values for Cramer's V showed moderate to strong associations of these domains with family function: social (0.453), cultural (0.382), and medical (0.304). Results also showed a significant association between the over-all adequacy of family resources based on the entire SCREEM-RES and family function based on the Family APGAR (chi square (2) = 16.157, exact p = 0.001; p = .002, FET). Cramer's V was 0.426 which indicates a strong association. For the analysis, all contingency tables had at least 80% of expected frequencies that were 5 or larger. There was no significant association found between family resources and caregiver strain based on MCSI, and between family function and caregiver strain.

The study did not find evidence for significant associations between caregiver strain and other demographic variables in the sample studied including: caregiver's age group (<20, 20-35, 36-50, >50), caregiver's educational attainment (elementary, high school, or college), caregiver's gender (male, female), relationship to the patient (mother, father, grandparent, aunt/uncle, others), the patient's age group (<6, 6-12, 13-19) and patient's gender (male, female). There was also no significant association found between the family caregiver's educational attainment and the reported adequacy of educational resources based on the subscale for educational resources.

### Exploratory Factor Analysis

Exploratory factor analysis of the 12 item family resources instrument was done using the simplified 2-point scoring system for each item. The sample size of this study was inadequate (Kaiser-Meyer-Olkin Measure of Sampling Adequacy at 0.640) for an actual factor analysis, but an exploratory factor analysis was done to examine the structure of relationships between the items of the family resources questionnaire. Varimax with Kaiser Normalization was used. Initial analysis showed that the scale is composed of 4 domains which accounted for 63.3% of variance. All items under the original social, cultural and religious domains formed the first factor. The remaining items in the economic, educational and medical domains formed the other three factors which directly corresponded to the original domains.

## Discussion

According to Smilkstein's cycle of family function [13], families faced with stressful life events may experience disequilibrium and their ability to recover from this is determined by the availability of family resources; inadequate resources would lead to terminal disequilibrium and adequate resources would direct them into functional equilibrium. This is also true with the ABC × Model of Family Stress [18,19] which states that the severity and duration of family disorganization during a crisis is determined by the level of family functioning, the perceived magnitude of the crisis and the amount of family resources. Families with inadequate resources are less likely to return to their former functionality. Those who are more prone to go into severe and prolonged crisis tend to experience more problems and stressors, lack coping resources, and be more dysfunctional.

Based on the results of the Family APGAR and Modified Caregiver Strain Index (MCSI) many Filipino families and family caregivers experience significant psychosocial distress following the diagnosis of childhood cancer. These families need appropriate psychosocial care and support to help them cope with the illness.

The study used a newly developed instrument, SCREEM-RES to assess family resources which was based on Smilkstein's SCREEM method of analysis of family resources [5,13]. The results of which revealed that economic resources were the most commonly cited as being inadequate, this is followed by inadequate access to medical care in the community, inadequate educational resources to understand disease, inadequate help from the community, and inadequate educational resources to learn to provide care. On the other hand, in decreasing order, the resources which were most commonly cited as being adequate were faith and religion, help from health care providers, help within the family, and cultural resources.

The Philippine government has low budget for health; a local television news program aired last August 12, 2011 that the administration only allocates Php 300.00 or USD 7.14 of its budget per annum for every Filipino's medical services. Most of the country's hospitals are privately-owned; moreover, its social insurance arm, the Philippine Health Insurance Corporation (PhilHealth) only gives health benefits to regular paying members. Thus, the nation's healthcare system could hardly help its sick people and their caregivers and this is much felt by cancer victims who shoulder much if not all of their treatments.

Many Filipino families, who are living in the Philippines, have limited economic or financial resources. Based on data from the National Statistics Office (2000), 70% of Filipino families can only afford to spend less than Php 2,200 (approximately 45 USD) for healthcare-related expenses every year [20]. Many low-income families who are being seen at the government hospital where this study was done, do not even have adequate financial resources to sustain their basic needs as most of them are minimum wage earners or unemployed thus they have little or no savings at all. Generally, their limited economic resources is related to the reported inadequacy and inaccessibility to medical care in the community. Aside from the families' inability to pay for their own medical care, many of their communities have also limited financial resources that could provide them health care. This condition led the private health care institutions and providers to avoid the poor communities and serve only the most progressive areas in the Philippines. These factors contribute to the serious imbalance in the distribution of medical care resources in the country which favors the most developed communities.

With regards to the report of inadequate educational resources, most of our respondents have reached high school and this should have allowed them to understand basic information about the illness and instructions on caregiving. This inadequacy may be due to other factors such as inadequate communication and information sharing between family caregivers and health care providers. This may also reflect the family caregiver's need for more counseling and guidance from healthcare providers.

Religious resources were the most commonly cited family resources which were adequate. Filipino families are known for being religious; religion and religious practices form a significant part of family life. They are usually readily available to Filipino families as churches, various religious institutions and religious groups are found in almost all Filipino communities. These resources are believed to be important in times of crisis; according to Martini [21], religion can be a source of strength for the family. He also stated that accessible religious support services and religious practices can decrease the stress and burden of caregiving.

Also important in times of crisis is social support. The amount of distress experienced by families and parents of children with cancer is related to the perception of and satisfaction with social support [22-26]. Results of the current study showed that social resources, particularly the help within the family, were cited as the next most adequate family resources. This is supported by the fact that in general, Filipinos are known to have close knit families, neighbors and friends in the community are usually ready to help the family in times of need.

Although many family caregivers had reported inadequate access to medical care in their community, majority of caregivers also reported that the help they receive from health care providers was adequate. Many families in this study have travelled long distances from their communities to seek consult and medical care which was not available or accessible in their own communities. The high percentage of caregivers who reported adequate help from health providers may have referred to the help that they were receiving from providers in the hospital where this study was done which has a well established supportive and palliative care program which helps patients and families, and not to the help from healthcare providers in their own communities.

Using the original 4-point scoring system for each item, calculated values of Cronbach's alpha, inter-item and item total correlation indicated that the set of questions for each 2-item domain and the entire 12-item questionnaire had good reliability. For a preliminary assessment of the SCREEM-RES, item scores were collapsed to form a 2-point scoring system. Using this scoring system, values of Cronbach's alpha, inter-item and item total correlation were slightly lower. However, reliability measures remained adequate for group analysis except for the medical domain scale. Exploratory factor analysis was also done on the items of the family resources questionnaire. Four factors were extracted: socio-cultural-religious factor (includes all items in the original social, cultural and religious domains), educational factor (items in the educational domain), economic factor (items in the economic domain), and medical (items in the medical domain). This factor structure is conceptually consistent with and supports the original SCREEM based six domain structure. Items in the original social, cultural and religious domains, which all loaded into the first factor, are all closely related to the socio-cultural aspects of family life. While the three other original domains directly corresponded to the three other factors extracted.

For this study, scores for each domain and the entire family resources instrument were grouped into dichotomous categories (adequate resources and inadequate resources). Standard recommended categories for the family APGAR and the MCSI were used for family function and family caregiver strain respectively. Results showed that there is evidence of significant association between adequacy of family resources and family function. However, the study did not find any significant association between family resources and caregiver strain, and between family function and caregiver strain. This may be partly explained by the fact that both the family resources questionnaire and the Family APGAR are family focused instruments, while the MCSI is focused on individual caregivers. Other factors may affect caregiver strain such as the caregiver's personal or internal resources and the patients' severity of the illness, degree of incapacitation and caregiving needs. A more intensive psychometric evaluation and evaluation of the SCREEM-RES were done, and will be presented in a succeeding paper [Medina M, Panganiban-Corales A, Nicodemus L and Ang A. 2010. Family Resources Study Part 2: Development and Psychometric Assessment of the SCREEM Family Resources Survey (SCREEM-RES). Unpublished].

The majority of the family caregivers in the study were young mothers who are more prone to experience significant stress and caregiver strain [27-29]. However, the current study was not able to show any significant association between caregiver strain and the caregiver's age, gender and relation to the child. There was also no evidence of significant association between caregiver strain and other demographic variables including the caregiver's educational attainment, and the patient's age and gender. Further studies are needed to determine the relationships between these demographic variables.

This study focused on family resources, family function and family caregiver strain; and the relationships between these factors. A better understanding of these factors and their relationships is important in the development of ways to determine the family's risk of going into severe crisis. It is also important in the development of appropriate and effective family interventions to help families who are at risk and families who are already in severe crisis. Moreover, this study provided initial evidences that the family resources instrument used in this study is a helpful, reliable and valid tool in the assessment of the adequacy of family resources, especially in times of severe stress and crisis.

## Study Limitations and Future Research Recommendations

The relative cost and time that has required the researchers to carry out this investigation was small using convenience sampling; it also enabled them to gather useful information that would have not been possible using probability sampling techniques. However, the subjects they used in this study may not have wholly represented the population of pediatric cancer caregivers; hence, it limited the ability of this paper to make generalizations about the entire population. It is therefore recommended that a randomized population be generated in a follow-up study so as to avoid under or over representation of this particular group of caregivers.

The cross-sectional study design used in this study found significant associations between family resources and family function, but the study design also limited the ability to establish causality. Future longitudinal studies may better describe the roles of family resources, family functioning and caregiver strain during the illness trajectory. Also, this survey is based on self-report, and may be prone to social desirability bias. Social desirability bias is a tendency of the respondents to reply in a manner that will be viewed favorably by others which leads to over reporting of good caregiver and family characteristics and under reporting of bad characteristics. Though most of the family caregivers were mothers, other relatives were also included in the study. Reliance on different family members may be a limitation considering that they may have different interactions with the child and possibly different emotional reactions to the child's illness.

The study was also limited by the availability of appropriate Filipino data collection tools and instruments. The lack of readily available Filipino psychosocial tools to assess the family was a significant limitation of the study. Future studies are warranted once more Filipino family assessment instruments become available. The family resources questionnaire used in this research, like the original SCREEM Method of Analysis by Smilkstein from which it is based, is only meant to do a general assessment of family resources. It is planned to be a generic instrument that can be used in different populations and settings. Assessment tools which are more caregiver focused, or more disease specific are also needed. For example, a more caregiver-specific tool can be developed by including variables that are more directly related to caregiver strain such as personal or internal resources, severity of patient's illness, and the patient's caregiving needs. Future studies should assess the relations between these variables to family resources and family function-dysfunction in order to comprehensively evaluate all possible family resources that could help families cope with crisis. A more intensive evaluation of the SCREEM-RES was done, and will be presented in a succeeding study (Medina, Panganiban-Corales, Nicodemus and Ang, 2010).

A follow up study using a bigger and more heterogeneous population is also recommended. These studies can further evaluate the reliability and validity of the SCREEM-RES. Furthermore, studies are needed to better determine cut off values for adequacy of family resources. Results of this study described the psychosocial needs of families and caregivers. These results support the use of family interventions to improve family resources, support family function, and ease caregiver strain.

## Conclusion

Many Filipino families of children with cancer have inadequate family resources, especially economic resources, to deal with medical crises; and many families are moderately or severely dysfunctional.

Many adult family caregivers are predisposed to caregiver strain or are already experiencing severe strain. Majority of Filipino family caregivers of children with cancer disclosed that they have inadequate economic resources. Many families also report inaccessibility to medical help in the community and insufficient education to understand and care for their patients. Most families acknowledged that their family's faith and religion is helpful; they also felt that help from within the family and health providers were adequate. In order to be able to provide appropriate care for families of patients with severe illnesses such as childhood cancer, physicians should regularly assess family function, family resources and the strain experienced by family caregivers. The SCREEM-RES questionnaire, which was used in this study, is a helpful and reliable instrument to assess the adequacy of family resources.

### Permission and Use of the Family Resources Questionnaire

Permission on the use of family resources questionnaire utilized in this study must be obtained from Manuel F. Medina, Jr. M.D. and the Section of Supportive Palliative and Hospice Medicine, Department of Family and Community Medicine, University of the Philippines - Philippine General Hospital, Manila. The content and format of this family resources questionnaire must not be modified or altered without the permission of the tool developer.

## List of Abbreviations

APGAR: is an acronym for Adaptability, Partnership, Growth, Affection, Resolve while; MCSI: stands for Modified Caregiver Strain Index; SCREEM-RES: stands for Social, Cultural, Religious, Economic, Educational, Medical Resources; SPHM: is an abbreviation for Supportive Palliative and Hospice Medicine.

## Competing interests

The authors declare that they have no competing interests.

## Authors' contributions

This document is based on a previous report for a research entitled "Testing the association of family resources with caregiver strain and family function among pediatric cancer patients using a novel SCREEM questionnaire" which was presented at the Annual Resident's Research Forum of the Department of Family and Community Medicine, University of the Philippines -Philippine General Hospital, October, 2010.

AC with the supervision of MM, was responsible for the overall preparation of the original research manuscript, including the analysis and write-up of the findings and discussion. The family resources questionnaire was developed by MM who also provided critical review of the original manuscript. The original manuscript was further edited, data analysis was checked and additional analysis was done by MM, resulting in the current manuscript. Both AC and MM read and approved the final manuscript.

## Authors' information

MM is a family medicine consultant at University of the Philippines-Philippine General Hospital and is the current head of the Section of Supportive Hospice and Palliative Medicine which provides palliative care to both adult and pediatric cancer patients in this institution. He has written various manuscripts on palliative care and has been active in teaching his residents and fellows in addressing psychosocial issues of patients.

AC is a newly certified family and community medicine specialist trained at the University of the Philippines-Philippine General Hospital. At the time of the conceptualization of her research as a requirement for graduation in residency training, she was inspired by the works of Dr. MM for children with cancer as she was rotating at the section of Supportive Palliative and Hospice Medicine where the latter is head thus both collaborated for the fulfillment of this study.
